# Consequences of early adverse rearing experience (EARE) on development: insights from non-human primate studies

**DOI:** 10.13918/j.issn.2095-8137.2017.002

**Published:** 2017-01-18

**Authors:** Bo Zhang

**Affiliations:** ^1^Yunnan Key Laboratory of Primate Biomedical Research, Kunming Yunnan 650500, China; ^2^Institute of Primate Translational Medicine, Kunming University of Science and Technology, Kunming Yunnan 650500, China; ^3^3National Institute of Health, Bethesda, Maryland, USA

**Keywords:** Early adverse rearing experience, Nonhuman primates

## Abstract

Early rearing experiences are important in one's whole life, whereas early adverse rearing experience (EARE) is usually related to various physical and mental disorders in later life. Although there were many studies on human and animals, regarding the effect of EARE on brain development, neuroendocrine systems, as well as the consequential mental disorders and behavioral abnormalities, the underlying mechanisms remain unclear. Due to the close genetic relationship and similarity in social organizations with humans, non-human primate (NHP) studies were performed for over 60 years. Various EARE models were developed to disrupt the early normal interactions between infants and mothers or peers. Those studies provided important insights of EARE induced effects on the physiological and behavioral systems of NHPs across life span, such as social behaviors (including disturbance behavior, social deficiency, sexual behavior, etc), learning and memory ability, brain structural and functional developments (including influences on neurons and glia cells, neuroendocrine systems, e.g., hypothalamic-pituitary-adrenal (HPA) axis, etc). In this review, the effects of EARE and the underlying epigenetic mechanisms were comprehensively summarized and the possibility of rehabilitation was discussed.

## INTRODUCTION

One of factors affecting life-long health of humans is the stability of early childhood, especially children's relationship with their mothers. John Bowlby's attachment theory suggests that individual's social relationship throughout life is influenced by the initial attachment with the mother (Bowlby, 1969). Attachment theory is a psychological, evolutionary and ethological theory concerning relationships among humans. Within the theory, attachment means an affectional bond or tie between an individual and an attachment figure (usually a caregiver). The core is that a child needs to build relationship with at least one primary caregiver to develop normal social and emotional behaviors. In many orphans, the lack of normal attachment to parents would cause behavioral and physical problems in childhood and possibly continuing throughout adult life (McEwen, 2003). Adults with adverse experience were more vulnerable to physical, psychosocial and mental disorders (Maughan & McCarthy, 1997; Pirkola et al., 2005). In human, early adverse rearing experience (EARE) usually refers to child abuse, which is a worldwide problem and is defined as neglect or physical, sexual or emotional mistreatment or abuse of children (Newton & Vandeven, 2009, 2010). Although human based studies revealed compelling associations between EARE and psychological outcomes, both retrospective and prospective studies showed their limits, e.g., inaccurate self-report due to biased or even false memory, failure in controlling accompanying environmental and genetic factors. Therefore, the long-term effects of EARE on subjects were usually not the direct consequences, but were inevitably intervened or masked by uncontrollable factors. However, experimental animals can be raised in laboratory environments, therefore allow researchers to carry out randomized prospective longitudinal studies, e.g., rigorously control or systematically manipulate early experiences throughout the entire period of investigation.

Rodents are easy to manipulate genetically, and the related studies indicate EARE as a developmental risk factor with profound, long-term effects on later life (Meaney, 2001; Pryce et al., 2005b; Sánchez et al., 2001). Whereas the high similarities of NHPs with humans make it irreplaceable in investigating the effects of EARE on physiological and behavioral development, e.g., NHPs and chimpanzees in specific, share over 90% and 98.8% genomes with human beings, respectively (Lovejoy, 1981). High similarities were found in both biological (Azmitia & Gannon, 1986; Uylings & van Eden, 1991) and socio-ecological aspects, e.g., social organizations and clear dominance hierarchies (Bailey & Aunger, 1990; DeVore, 1990; Wright, 1990). The phenomenon that in NHPs, 2%-10% of infants were physically abused or neglected by their mothers in group-living conditions, allow the possible screening of natural child abuse models (Maestripieri & Carroll, 1998; Maestripieri et al., 1997). Moreover, like humans NHPs has prolonged postnatal period of maturation during which mother-infant relationship and neural system development can be influenced by environment and early life experience (Levine & Wiener, 1988; Suomi, 2005). 

Harlow (Harlow & Harlow, 1965) introduced the concept of affectional systems to characterize the relationships in the social groups of primates, and five distinct affectional systems were described, including the infant-mother affectional system, the maternal affectional system, the age-mate/peer affectional system, the heterosexual affectional system and the paternal affectional system. The infant-mother and the maternal affectional systems in Harlow's affectional systems are similar to Bowlby's concept of mother-infant attachment theory in humans. In normal living group, most monkey infants virtually spend all of their initial days or weeks of life clinging with their biological mothers, ventral to ventral, during which, specific and strong attachment bonds are built. When about 2-month old, infants begin to explore the physical and social environment, spending increasing amount of time participating social interactions, especially playing with peers. From 6-month of age until puberty, playing with peers becomes the major social activity (Hinde & Spencer-Booth, 1967; Suomi, 1997, 2005). In fact, the infant and juvenile monkeys always maintain a close social relationship with their mothers, while the mother plays the role of protector especially under stressful situations, and mentor in teaching developing appropriate social behaviors. Accordingly, the studies regarding EARE usually involve disruption of the normal infant-mother relationships, by maternal deprivation of newborns, maternal separation or induced stress on older infants and juvenile monkeys. Although some epidemiological studies in humans suggest possible direct relationships between EARE and abnormal behaviors in later life, no solid evidence was raised to prove the precise impact of childhood adversities on psychiatric disorders (Benjet, 2010; Bick & Nelson, 2016; Gershon et al., 2013; Kessler et al., 1997; Kessler & Wang, 2008; Klein et al., 2013; Sheridan et al., 2010). The over 60 years NHP studies shed lights on the understanding of the influences of EARE on physiological and behavioral development, including social behaviors (e.g., disturbance behavior, social deficiency, sexual behavior, etc), learning and memory ability, brain structural and functional development (e.g., development of neurons and glia cells, neuroendocrine dysregulation, etc). In this review, the previous findings on EARE were systematically summarized, and the underlying epigenetic mechanisms and the potential methods of rehabilitation were thoroughly discussed. 

## EARE MODELS IN NON-HUMAN PRIMATES

Controlled rearing conditions in standard laboratory settings are designed to simulate natural environments. The infants are reared by their mothers and live in a group consisted of other infants, juveniles and adults, allowing infants to be exposed to complex social interactions. In abnormal rearing conditions, the mother deprivation method is applied. The newborn is taken away from their mothers at birth and is reared in incubators with regular medical attention and laboratory nursery. A period of time (usually 1-month) later, when able to feed themselves, infants are moved to other rearing conditions depending on aims of research, e.g., be reared alone in social isolation condition, with nursery/peers of the same age in nursery/peer rearing condition, with a surrogate in surrogate mother/foster rearing condition, etc. They could also be separated from mothers at later time for once (temporary maternal separations) or several times (repetitive maternal separations); or even though staying with their mother all the time, but still suffer from EARE (maternal neglect).

### Social isolation

Social isolation (including total and partial social isolation) is initially described in early 1960s by Harlow and his colleagues, and has been used ever since to raise monkeys in simulating social behavior deficits in humans (Table 1). In total isolation, the infant is reared in a cage alone without any auditory, visual, olfactory and tactile contact with conspecifics, including mothers, peers and other monkeys (Baysinger et al., 1972; Harlow & Harlow, 1962; Harlow et al., 1964, 1965). In partial social isolation, although infants are separately caged from their mothers, peers, and social groups, they have auditory, visual, and olfactory but not tactile contact with their conspecifics (Cross & Harlow, 1965; Mason & Sponholz, 1963; Struble & Riesen, 1978; Suomi et al., 1971). These early studies by Harlow and his colleagues, especially their extreme manipulations, including total isolation, "pit of despair" and "rape rack" devices, were controversial and were most likely forbidden to perform due to ethical issues. In 1950s, many researchers assumed that the only necessity of mother was supplying food to infants, whereas excessive intimacy between mother and infant would hinder the growth of infant, or even induce over dependence in adulthood. Harlow disagreed with the viewpoints; performed a series of isolation studies on primates to prove that to acquire necessary social skills, to obtain both physically and psychologically healthy development, infants need mothers' affection, as well as normal social interaction and emotional relationship with peers. However, their intention was to prove the essential role of mother's love to infants, in the form of her availability all the time, her physical touching, caring and protection, which was an obvious fact to us today without any necessity to prove. 

**Table 1:** Early adverse rearing experience (EARE) methods

Paradigms		Description			References
Social Isolation	Total	Infants are reared in a cage alone at birth, no any auditory, visual, olfactory and tactile contact with conspecifics is allowed			Baysinger et al., 1972; Harlow et al., 1965; Harlow & Harlow, 1962; Harlow et al., 1964
	Partial	Infants are separately caged at birth, reared with auditory, visual, and olfactory, but not tactile contact with conspecifics			Cross & Harlow, 1965; Mason & Sponholz, 1963; Struble & Riesen, 1978; Suomi et al., 1971
Maternal separation	Permanent	Peer-rearing	Continuous	Infants are reared by pairs throughout development	Chamove et al., 1973; Erwin et al., 1973; Sackett, 1967; Worlein & Sackett, 1997
			Intermittent	Peers are allowed to contact with each other for a limited period of time and then infants are housed singly during the rest of the time	Rommeck et al., 2009b
			Rotational	Infants are continuously housed with different peers	Novak & Sackett, 1997; Rommeck et al., 2009b
		Surrogate mothers rearing (SMR)	Inanimate objects are placed into the cage as an artificial surrogate mother		Capitanio & Mason, 2000; Dettmer et al., 2008; Schneider & Suomi, 1992; Suomi, 1973
		Surrogate-peer rearing (SPR)	Combination of SMR and PR		Bastian et al., 2003; Lutz et al., 2007; Meyer et al., 1975
	Temporary	One time	Infants are taken away from their mothers at later stages of life for a period of time, followed by mother-infant reunion		Hinde & Mcginnis, 1977; Hinde et al., 1966; Kaufman & Rosenblum, 1967; Seay et al., 1962; Spencer-Booth & Hinde, 1971
		Repetitive	Repeatedly separating infants from their natal group for relatively short periods of time, followed by repeated reunions		Clarke et al., 1998; Dettling et al., 2002a, b ; Levine & Mody, 2003; Sánchez et al., 2005 ; Suomi et al., 1983
Maternal neglect			Infant-mother was confronted with various foraging conditions to induce different levels of stress in the mother		Andrews & Rosenblum, 1991; Coplan et al., 1996; Rosenblum & Andrews, 1994; Rosenblum & Paully, 1984

However, although isolation models are important in highlighting the devastating consequences of maternal deprivation, the extreme manipulations could induce severe cognitive and emotional deficits, or even self-injurious behaviors, which are very difficult to remediate in primates. Therefore, less severe rearing conditions were developed afterwards at least partially due to ethical considerations. 

### Maternal separations

Peer-rearing (PR) (or nursery rearing, NR) (including continuous pair rearing, intermittent and rotational peer rearing) is another widely used rearing condition, in which infants were reared together with peers of the same age (Chamove et al., 1973; Erwin et al., 1973; Sackett, 1967; Worlein & Sackett, 1997) (Table 1). In continuous pair rearing condition, infants are usually reared by pairs throughout development (Chamove et al., 1973; Fekete et al., 2000; Hotchkiss & Paule, 2003; Novak & Sackett, 1997). Intermittent peer rearing allow peers to contact with each other for a limited period of time, and then infants are housed singly during the rest of the time (Rommeck et al., 2009b). Within the rotational peer rearing condition, infants are continuously peer housed with different infant partners (Novak & Sackett, 1997; Rommeck et al., 2009b). Previous study showed that continuous rotational pairing induces a behavioral profile quite similar with that of mother rearing in socially complex environment (Rommeck et al., 2011). Compared with social isolation, PR is less severe and thus more widely used in recent NHP EARE studies. Surrogate mothers rearing (SMR) is another early rearing method, in which inanimate object is placed into the cage as an artificial surrogate mother (Capitanio & Mason, 2000; Dettmer et al., 2008; Eastman & Mason, 1975; Harlow, 1958; Harlow & Zimmermann, 1959; Hennessy & Kaplan, 1982; Kaplan, 1974; Mason & Berkson, 1975; Roy et al., 1978; Schneider & Suomi, 1992; Suomi, 1973). Infants could quickly develop attachment with surrogate mothers, and some studies indicated that the infants usually preferred cloth surrogate mothers than wired ones (Harlow, 1958; Harlow & Zimmermann, 1959). Previous study reported that surrogate mothers could affect the behaviors of infants, and different characters of surrogate mothers such as mobility and orientation had different influences (Dettmer et al., 2008). Surrogate-peer rearing (SPR) method is a combination of SMR and PR, in which the infants are reared with inanimate surrogate mothers (SMR condition) during the initial several months of life, and then are allowed to have peer interactions for a limited period of time (PR condition) (Bastian et al., 2003; Lutz et al., 2007; Meyer et al., 1975). Comparing with permanent removal of the mother, infants are not separated from their mothers right away at born in temporary maternal separations, but after a period of time usually several hours, days or weeks, following by mother-infant reunion (Hinde et al., 1966; Hinde & McGinnis, 1977; Kaufman & Rosenblum, 1967; Seay et al., 1962; Spencer-Booth & Hinde, 1971). Temporary maternal separation usually contains a one-time separation although different time delay could be adopted. A modified version of one-time separation is repetitive mother-infant separation, in which infants are separated from and reunited with their natal group repeatedly for relatively short periods of time (Clarke et al., 1998; Dettling et al., 2002b; Levine & Mody, 2003; Sánchez et al., 2005; Suomi et al., 1983). The impact of these procedures appeared to be further intensified if the separations were unpredictable (Levine, 2000; Sánchez et al., 2005). Unlike social isolation, maternal separation adopted relatively mild manipulations, the presence of surrogate mothers and the opportunity of direct contact with mothers and peers added social complexity to the infants' living environment, therefore could avoid severe social and emotional deficits associated with mothers' absence.

### Maternal neglect

Compared with isolation and maternal separation methods described above, maternal abuse and neglect during early life are more common in humans, therefore are more widely used on NHPs to study adult mood and anxiety disorders. In NHP maternal neglect models, in order to induce stress in the mother, infant mothers are confronted with various foraging conditions, such as variable/unpredictable foraging demand (VFD), consistently low foraging demand (LFD) and consistently high (but predictable) foraging demand (HFD). Mothers in LFD condition have easy access to food while those in high foraging demand have to work hard to get food (Andrews & Rosenblum, 1991; Coplan et al., 1996; Rosenblum & Andrews, 1994; Rosenblum & Paully, 1984). The advantage of this model is that even though infants are still in adverse situation, the severe adverse experience of mother and peer deprivation can be avoided. In addition, other rearing strategies are applied in this model, i.e., infants were reared by a female which was not their biological mother (Maestripieri, 2005; Novak & Suomi, 1991); infants were housed with non-reproductive female adults (Champoux et al., 1989b). 

## EARE EFFECTS

Although partial social isolation tends to induce less severe defects than total social isolation, the expression of behavior defects is similar. Isolated monkeys reared without exposure to companions during early life, especially the first 6 months, develop a pervasive pattern of abnormalities referred to as the isolation syndrome. Mason (Mason, 1968) summarized the syndrome under four headings: (1) abnormal posturing and movements, such as rocking; (2) motivational disturbances, such as excessive fearfulness or arousal; (3) poor integration of motor patterns, such as inadequate sexual behavior; (4) deficiencies in social communication, such as failure to withdraw after being threatened by an aggressing animal. In this section, the effects of EARE on social behaviors, learning and memory ability, brain structural and functional developments, including influences on neurons and glia cells, neuroendocrine dysregulation, especially stress related HPA axis will be reviewed. 

### Social behavior

Effects of EARE on social behaviors are detailed in Table 2.

**Table 2:** Effects of EARE on social behaviors

Behavior types	Behavior descriptions
Stereotypic behaviors	Whole-body stereotypes (e.g., rocking, pacing, bouncing, swing, and back-flipping)
	Self-directed stereotypes (e.g., saluting, digit-sucking, self-clutching, self-clasping, eye-poking, eye-covering and hair-pulling)
Self-directed behaviors	Self-manipulation, self-scratching, self-grasping, self-rubbing
	Self-injurious behavior (SIB)
Aggression	Less aggression during infancy and more aggression during later life
Affiliative behavior	Tend to show more affiliative behavior during infancy but less affiliative behavior during adulthood
Social and environmental exploration	Decreased social and environmental exploration
Social dominance	Tend to show low dominance rank
Sexual behaviors	Less and abnormal sexual behaviors
Others	Polyphagia and polydipsia

### Disturbance behavior

Monkeys exposed to adverse early experience tended to show more disturbance behaviors, such as stereotypic and self-directed behaviors, motivational disturbances and social deficiency. The isolated monkeys appeared to show more disturbance behaviors, including crouching, clutching, rocking, pacing, flipping, hugging, clasping, thumb-sucking (Harlow & Harlow, 1962; Harlow & Suomi, 1971a; Mason & Sponholz, 1963; Mitchell, 1968; Suomi et al., 1971). Among these monkeys, some abnormal movements, such as rocking and self-grasping, could present very early in their lives, even at the first month (Baysinger et al., 1972). Additionally, some of these behaviors could turn into stereotypic behaviors, including repetitive movements or postures, as well as ritualized movements, and could be divided into whole-body stereotypes (e.g., rocking, pacing, bouncing, swing, and back-flipping), self-directed stereotypes (e.g., saluting, digit-sucking, self-clutching, self-clasping, eye-poking, eye-covering and hair-pulling) and other idiosyncratic behaviors (e.g., teeth grinding, head tossing, or making noise by blowing air into the cheeks). It was reported that whole-body stereotypes were much more common than self-directed stereotypes (Lutz et al., 2003). Previous studies indicated that isolated monkeys showed more repetitive whole-body stereotypes (Mitchell, 1968), while PR monkeys showed more self-directed stereotypes (Lutz et al., 2003; Suomi et al., 1971).

EARE exposed monkeys tended to show more self-directed behaviors. Isolated monkeys showed self-manipulation, self-scratching, self-grasping, self-rubbing, and autoeroticism while in isolation (Baysinger et al., 1972), or showed remarkable increases in self-clasping soon after removal from isolation (Harlow et al., 1965; Suomi et al., 1974), or self-clutching after surrogate mother removing (Harlow & Zimmermann, 1959). PR reared infants and juvenile monkeys showed increased self-stimulation behaviors, including self-sucking, self-clinging, self-clasping and other self-directed behaviors (Champoux et al., 1991; Lutz et al., 2003; Suomi et al., 1971). Moreover, short-term stress by temporary physical restrictions could also induce significant increases in self-clasping and huddling behaviors when the infants returned to their home cages (Harlow & Suomi, 1971a). Those self-directed behaviors often turned into self-injurious behavior (SIB), with males showing a much higher level of vulnerability than females (Cross & Harlow, 1965; Gluck & Sackett, 1974; Lutz et al., 2003; Suomi et al., 1971), and PR monkeys usually much more vulnerable than MR monkeys (Rommeck et al., 2009a). Surrogate mothers appeared to provide a certain degree of contact acceptability, security and trust sufficient for isolated monkeys to suppress existing self-directed disturbance activity, and to initiate crude social interactions with other isolated monkeys (Harlow & Suomi, 1971b). However, Lutz et al. (Lutz et al., 2007) reported that SPR monkeys showed significantly more self-biting comparing to PR and MR reared animals, and it was suggested that surrogate rearing in combination with lower levels of social contact during play may be risk factors for the later development of self-biting behavior. Actually, self-directed behaviors were hypothesized to result from the redirection of normal social behaviors toward one's own body and were suggested to be symptoms of some mental diseases (Goosen, 1981; Mason & Berkson, 1975). These findings indicate that EARE exposed monkeys could be used as an ideal model of related human mental disorders from behavioral perspective.

### Social deficiency

In natural environments, infants and juvenile monkeys are supposed to be more active in joining the social play with peers, but monkeys exposed to EARE show decreased social playing. Isolated monkeys showed less (Harlow et al., 1965; Mitchell, 1968), or even no contact playing at all (Harlow et al., 1965). Pair and peer reared infants (Chamove et al., 1973), VFD reared infants (Andrews & Rosenblum, 1991; Rosenblum & Paully, 1984), repeated parental deprivation infants (Dettling et al., 2002b; Levine & Mody, 2003) all showed less social playing compared with MR infants. Lack of sufficient social interaction led to the fact that EARE exposed monkeys could not successfully adapt to living in a large social group (Griffin & Harlow, 1966; Harlow & Harlow, 1962; Mason & Sponholz, 1963; Ruppenthal et al., 1991). Not only social interaction, studies also showed decreased environmental exploration in isolated monkeys (Griffin & Harlow, 1966; Mason & Sponholz, 1963; Mitchell, 1968), VFD and PR monkeys (Rosenblum & Paully, 1984; Ruppenthal et al., 1991). Another major index of exploratory behavior is locomotor activity, while some NHP studies showed less locomotion in isolated adults (Harlow & Suomi, 1971a; Mason & Sponholz, 1963; Mitchell, 1968) and PR infants (Feng et al., 2011), others found no differences in PR adults (Winslow et al., 2003), or even higher activity levels in PR infants during the first month after isolation (Champoux et al., 1991). Therefore, there was no agreed tendency of EARE influence on locomotor activity in monkeys, making it an invalid measure of exploratory behavior if used alone (Wright, 1983). 

Another domain of EARE induced social deficiency is social dominance. In monkey society, social dominance is a complex phenomenon mediated by different mechanisms and various factors such as kinship, age, sex, and physical factors like body weight, appearance and health (Bernstein & Cooper, 1999; Bernstein & Mason, 1963; Morgan et al., 2000; Sprague, 1998; Takahashi, 2002). Kinship seemed to be the major factor in determining dominant rank at least until puberty (Koford, 1963; Koyama, 1967), but became weaker during the development (Bernstein & Williams, 1983). Both dominance formation and maintenance among males in a living group are usually achieved by aggressive behavior such as fighting, with the stronger and more aggressive subjects winning and thus becoming dominant. However, appropriate use of aggression is critical for both acquiring and maintaining social status, as overly aggressive monkeys may risk social ostracism from their conspecifics. Moreover, aggressive behavior was not indispensable to obtain and keep dominance status and dominance sustained without aggression was more stable than that formed on the basis of aggression (Fonberg, 1988). 

Monkeys exposed to EARE tended to show less aggression during infancy (Chamove et al., 1973; Harlow et al., 1965), and more aggression during later life (Chamove et al., 1973; Mitchell, 1968; Suomi et al., 1974; Winslow et al., 2003). The aggressive monkeys exposed to EARE may repeatedly attack a helpless infant or attempt to attack a dominant male, while infant-directed aggression is abnormal adult-directed aggression is both abnormal and suicidal (Chamove et al., 1973; Mitchell, 1968; Suomi et al., 1974; Winslow et al., 2003). On the other hand, studies showed EARE exposed monkeys showed heightened fear in all age stages (Champoux et al., 1991; Dettling et al., 2002b; Levine & Mody, 2003; Mitchell, 1968). It seems that EARE makes monkeys more emotional in two opposite directions, both aggression and fear. In addition to aggression, affiliative behavior, such as grooming and proximity, is also important in establishing and maintaining alliances and reinforcing the dominance hierarchy. Affiliative behavior was suggested to be more positively related to dominance rank than kinship in Japanese monkeys (Singh et al., 1992). On the contrary to aggression, EARE exposed monkeys showed more affiliative behavior during infancy (Chamove et al., 1973; Rosenblum & Paully, 1984; Ruppenthal et al., 1991), but less affiliative behavior during adulthood (Kraemer & McKinney, 1979; Rosenblum & Paully, 1984; Winslow et al., 2003). With more aggressive and less affiliative behavior which both contribute to acquiring and reinforcing social dominance, EARE exposed adult monkeys are supposed to have low social dominant rank in a living group, and studies indeed indicated that both isolated and PR adult monkeys showed low social dominance (Kraemer & McKinney, 1979; Mitchell, 1968; Ruppenthal et al., 1991). 

### Sexual behavior

Monkeys exposed to EARE demonstrated less or abnormal sexual behaviors (Chamove et al., 1973; Harlow et al., 1966; Harlow, 1962; Harlow et al., 1965; Mitchell, 1968). Abnormal sexual behaviors (abortive mount) is defined as any improperly oriented mount, accompanied by pelvic thrusting including standing-to-head, standing-to-side and ventral lie-on (Wallen et al., 1981). Males usually were not mount properly as they engaged in varied but misplaced heterosexual efforts, while females were not maintain the sexual present (stood quadripedally with the perineal area directed towards the recipient) or turned their bodies when mounted. Mount behavior includes no-foot-clasp mount and foot-clasp mount, which could be differentially affect by different EARE. Males with short access periods with peers (0.5 h) rarely or never foot-clasp-mounted peers, while those given 24 h access regularly foot-clasp-mounted peers (Wallen et al., 1981). Isosexually reared males showed less foot-clasp mounting and more presenting than heterosexual males, while conversely, isosexually reared females showed statistically more mounting and less presenting than heterosexual females (Goldfoot et al., 1984). Moreover, females exposed to EARE also showed abnormal maternal behaviors, in a way that those never experienced mother caring not only were unable to exhibit caring to their own offspring, but also far more likely to display inadequate, abusive or neglectful behavior toward their offspring (Bridges et al., 2008; Champoux et al., 1992; Harlow & Suomi, 1971b; Seay et al., 1964; Suomi, 1978; Suomi et al., 1974; Suomi & Ripp, 1983), consistent with human findings showing abusive behavior appeared to be transmitted across generations (Roustit et al., 2009).

Primate studies also showed other EARE induced behavioral effects besides listed above, including polyphagia and polydipsia in isolated adults (Miller et al., 1969), more vulnerable to excessive alcohol consumption (Fahlke et al., 2000; Higley et al., 1991) and elevated response to both aversive and rewarding stimuli (Nelson et al., 2009) in PR monkeys and abnormal sleep rhythmicity (Barrett et al., 2009; Boccia et al., 1989; Kaemingk & Reite, 1987; Reite et al., 1974; Reite & Short, 1978). An interesting research showed EARE significantly influenced the development of lateralisation, as PR monkeys demonstrated greater left-hand bias compared to MR reared monkeys (Bennett et al., 2008). Despite of EARE effects described above, recent research suggested that modern PR practices might not result in inevitable perturbations in aggressive, rank-related, sexual, and emotional behavior in rhesus monkeys (Bauer & Baker, 2016). 

### Learning and memory

Early primate studies showed EARE exposed adults performed adequately on simple discriminations or delayed-response (Gluck et al., 1973), but showed impairments in certain complex tasks such as those requiring engaging working memory with dynamic rules or delays or response inhibition (Beauchamp & Gluck, 1988; Beauchamp et al., 1991; Gluck et al., 1973; Gluck & Sackett, 1976; Sánchez et al., 1998). These results were obtained mostly from adult monkeys separated from their mothers at birth and reared in total isolation for 9-12 months. PR reared juvenile monkeys also showed cognitive deficits, they had more difficulty acquiring the delayed non-matching to sample (DNMS) task and were also impaired in object but not spatial reversal learning (Sánchez et al., 1998). Moreover, even brief social isolation impaired performance in a multiple video-task assessment in adult rhesus monkeys (Washburn & Rumbaugh, 1991) and impaired reversal learning and behavioral inhibition in adult marmosets (Pryce et al., 2004a, b ). These results were consistent with the results of human studies, which showed the post institutionalized children (Bauer et al., 2009) and childhood exposed to neglect and abuse (Majer et al., 2010) were associated with impaired learning and memory during adulthood. Although those studies revealed EARE induced impairment of learning and memory ability in a task dependent way in adult monkeys, other primate studies indicate exposure to mild early life stress improves prefrontal dependent response inhibition in primates, suggesting its beneficial effect on cognitive control (Parker et al., 2005, 2012).

### Brain structure and function

The first documentation of the effects of negative early experiences on monkey brain was provided by Martin et al. (1991), which showed significant alterations in the chemo architecture of the striatum 19-24 years after social deprivation. Additionally, Siegel et al. (1993) demonstrated that early social deprivation resulted in an increase in the amount of non-phosphorylated neurofilament protein in hippocampal dentate gyrus granule cells in rhesus monkeys. Further studies showed structure and function changes in many brain regions including amygdala, hippocampus, prefrontal cortex (PFC), anterior cingulate cortex (ACC), corpus callosum and cerebellum etc, both in humans and animals exposed to EARE (Andersen, 2015; Bick & Nelson, 2016; Gilmer & McKinney, 2003; Gorman et al., 2002; Hart & Rubia, 2012; Korosi et al., 2012; McEwen, 2003; Worlein, 2014)(Table 3).

**Table 3:** Effects of EARE on brain structure and function

	Outcomes		Human Studies	Primate Studies
Amygdala	Children	No significant volumes changes	De Bellis et al., 2001; De Brito et al., 2013; Hanson et al., 2010; Woon & Hedges, 2008	No significant volume changes (Howell et al., 2014)
		Larger volume and elevated response	Lupien et al., 2011; Mehta et al., 2009; Tottenham et al., 2010	Decreased SERT binding potential (Ichise et al., 2006)
		Decreased volume	Edmiston et al., 2011; Hanson et al., 2015; Luby et al., 2013	Differential expression of one gene GUCY1A3 (Sabatini et al., 2007)
	Adults	No significant volume changes	Bremner et al., 1997; Cohen et al., 2006	
		Larger volume	Evans et al., 2016; Lyons-Ruth et al., 2016	
		Elevated activity	Casement et al., 2014; Javanbakht et al., 2015; Kim et al., 2013	
Hippocampus	Children	Decreased volume	Edmiston et al., 2011; Hanson et al., 2015; Luby et al., 2013	No significant volume change (Law et al., 2009a, b; Sánchez et al., 1998; Spinelli et al., 2009)
		No significant volume change	Carrion et al., 2001; De Bellis et al., 2001; De Bellis et al., 1999; De Bellis et al., 2002; Mehta et al., 2009; Tottenham et al., 2010 ; Woon & Hedges, 2008	
	Adults	Decreased volume	Bremner et al., 1997; Cohen et al., 2006; Stein et al., 1997 ; Woon & Hedges, 2008	
Prefrontal cortex (PFC)	Children	No significant volume changes	De Bellis et al., 1999	Greater enlarged medial prefrontal cortex (mPFC) size (Spinelli et al., 2009)
		Decreased volume	De Bellis et al., 2002; Edmiston et al., 2011; Hanson et al., 2010; Morey et al., 2016; Thomaes et al., 2010	
		Larger volume	Carrion et al., 2009; Richert et al., 2006	
	Adults	Decreased volume	Tomoda et al., 2009; van Harmelen et al., 2010	
		Reduced activity	Casement et al., 2015; Kim et al., 2013; Romens et al., 2015; Schweizer et al., 2016	
		Increased response	Casement et al., 2014; Javanbakht et al., 2015; Jedd et al., 2015; Wang et al., 2016; White et al., 2015	

### Amygdala

Amygdala is a group of almond-shaped nuclei located deep within the medial temporal lobes of the brain in complex vertebrates. It was considered as the emotion center and responsible for emotion reactions like reward, fear and anxiety (Davis, 1992; Gallagher & Chiba, 1996; Ledoux, 2003; Phelps, 2006). Rodent studies showed acceleration of amygdala development in early weaning rodents (Kikusui & Mori, 2009; Ono et al., 2008). The limited amount of primate studies found no significant amygdala volume changes (Howell et al., 2014), but functional changes including decreased SERT binding potential (Ichise et al., 2006) and differential expression of one gene GUCY1A3 (Sabatini et al., 2007) in amygdala of EARE exposed monkeys. However, human studies in maltreated children showed contrary results, with some studies found no volume changes (De Bellis et al., 2001; De Brito et al., 2013; Hanson et al., 2010; Woon & Hedges, 2008), while others revealed decreased volume (Edmiston et al., 2011; Hanson et al., 2015; Luby et al., 2013) or greater volume and elevated response (Lupien et al., 2011; Mehta et al., 2009; Tottenham et al., 2010). Furthermore, those studies found greater volume and elevated response of amygdala (Mehta et al., 2009; Tottenham et al., 2010) were performed several years after the institutionalized children adopted by high socio-economic status families. These data suggested that EARE modified amygdala changes was resistant to recovery, and it was consistent with primate research that suggested abnormal behaviors was resistant to environmental enrichment treatments (Lutz et al., 2004; Lutz & Novak, 2005; Novak et al., 1998; Rommeck et al., 2009a). Similarly, in adults exposed to EARE some studies found no significant changes of amygdala volume (Bremner et al., 1997; Cohen et al., 2006), while others found larger volume (Evans et al., 2016; Lyons-Ruth et al., 2016), interrupted regulation of negative emotion (Kim et al., 2013), increased response to potential rewards (Casement et al., 2014), elevated amygdala responses to threat but not happy faces (Javanbakht et al., 2015). In addition to amygdala structure and activity changes, its connectivity with other brain regions was also affected (Barch et al., 2016; Jedd et al., 2015). Despite those controversial results, the influence of EARE on emotion such as the elevated response to emotion stimuli both in human and primates (Casement et al., 2014; Javanbakht et al., 2015; Nelson et al., 2009) should be mainly achieved through its influence on amygdala.

### Hippocampus

Hippocampus, a major component of the brains located inside the medial temporal lobe and beneath the cortical surface, is involved in episodic, declarative, contextual, and spatial learning and memory, as well as being a component in the control of autonomic and vegetative functions (Buckley, 2005; Eichenbaum, 2001; Eichenbaum et al., 1992, 1996; Manns & Eichenbaum, 2006; Opitz, 2014; Shohamy & Turk-Browne, 2013). In human studies, EARE induced significant reduction of hippocampal volume was an consistent finding in adults (Bremner et al., 1997; Cohen et al., 2006; Hart & Rubia, 2012; McCrory et al., 2011; Stein et al., 1997; Woon & Hedges, 2008). However, children and adolescents studies showed inconsistent results, with few found decreased volume (Edmiston et al., 2011; Hanson et al., 2015; Luby et al., 2013), while most found no significant change (Carrion et al., 2001; De Bellis et al., 2001, 1999, 2002; Mehta et al., 2009; Tottenham et al., 2010; Woon & Hedges, 2008). Primate studies also found no significant hippocampal volume change in PR (Sánchez et al., 1998; Spinelli et al., 2009) and repeated mother deprived (Law et al., 2009b) juvenile monkeys, suggesting changes of hippocampus seemed to happen later in life compared to early life amygdala changes. Two possible explanations could account for the discrepancy of children and adult findings. Firstly, that might due to the fact that the hippocampus develops mainly in the first years of life, therefore less affected by exposure to adversity in childhood and adolescence (Houston et al., 2014; Lenroot & Giedd, 2006; Richards & Xie, 2015). Another possibility is that EARE might not have an immediate effect on the hippocampus but induced changes over time, and long-term effects of EARE exposure may be delayed and became manifest only in later phases of development when the vulnerable brain reaches maturation (Andersen & Teicher, 2004; Brunson et al., 2005; Gluckman & Hanson, 2004; Gluckman et al., 2007; Sapolsky et al., 1985). Moreover, human studies found interesting results concerned with influence of EARE exposure on structure and activity of hippocampus and amygdala, with decreased hippocampal volume and activity in humans exposed to adulthood stress (Bremner et al., 2007; Lupien et al., 2007; Rauch et al., 2000) and adults experiencing EARE (Bremner et al., 1997; Cohen et al., 2006; Stein et al., 1997; Woon & Hedges, 2008), while increased amygdala volume and activity in humans exposed to adulthood stress (Bremner et al., 2007; Lupien et al., 2007; Rauch et al., 2000) and adults experiencing EARE (Mehta et al., 2009; Tottenham et al., 2010). Although the biological mechanism and meaning of this phenomenon remains unclear, that might contribute to or even be the direct reason for the impaired learning and memory ability (decreased hippocampal volume and activity related) and elevated response to emotional stimuli (increased amygdala volume and activity related) described above.

### Prefrontal cortex

The prefrontal cortex (PFC) is the anterior part of the frontal lobes of the brain and implicated in planning complex cognitive behaviors, personality expression, decision making and moderating correct social behavior. Children and adolescents studies showed inconsistent results of EARE induced PFC structural changes, with findings of either no significant differences (De Bellis et al., 1999), or significantly smaller volume (De Bellis et al., 2002; Edmiston et al., 2011; Hanson et al., 2010; Morey et al., 2016; Thomaes et al., 2010) or significantly larger volume (Carrion et al., 2009; Richert et al., 2006). In contrast, decreased PFC volume in adults exposed to childhood maltreatment was a consistent finding (Tomoda et al., 2009; van Harmelen et al., 2010). That might due to the fact that PFC continues to develop during adolescence (Houston et al., 2014; Lenroot & Giedd, 2006; Richards & Xie, 2015), therefore might be particularly vulnerable to the effects of stress during adolescence. In addition to the structural changes, EARE could also induce PFC functional changes, with some human adults exposed to EARE showing reduced prefrontal cortex activity during monetary reward anticipation and emotion regulation (Casement et al., 2015; Kim et al., 2013; Romens et al., 2015; Schweizer et al., 2016), while others showing increased response to potential rewards and threatening faces and in passive viewing conditions (Casement et al., 2014; Javanbakht et al., 2015; Jedd et al., 2015; Wang et al., 2016; White et al., 2015). One primate report indicated PR juvenile monkeys showed greater enlarged medial prefrontal cortex (mPFC) size (Spinelli et al., 2009). Moreover, both rodent and primate studies revealed the direct underlying epigenetic mechanisms of EARE on PFC through influencing differential gene expression, histone acetylation and DNA methylation (Blaze et al., 2015a; Provençal et al., 2012; Wall et al., 2012). Studies regarding EARE effects on PFC in primates are rare, and further investigations are necessary. 

### Other brain regions

The anterior cingulate cortex (ACC) is the frontal part of the cingulate cortex, and appears to play a role in a wide variety of rational cognitive functions, such as reward anticipation, decision-making, empathy and emotion (Devinsky et al., 1995; Drevets et al., 2008). It can be divided anatomically into dorsal and ventral components, with dorsal part connected with PFC making its involvement in cognition possible, and the ventral part connected with amygdala making its involvement in emotion possible (Bush et al., 2000; Morecraft et al., 2007). Human studies showed reduced volume of adult ACC in people with mood disorders (Botteron et al., 2002; Drevets et al., 1997; Yamasue et al., 2003), adults exposed to early life stress (ELS) (Cohen et al., 2006) and abuse-related Posttraumatic stress disorder (PTSD) (Kitayama et al., 2006; Thomaes et al., 2010) and major depressive disorder (Treadway et al., 2009). On the contrary, a primate study found enlarged ACC in PR juvenile monkeys (Spinelli et al., 2009). Moreover, an epigenetic study showed parental separations in infant marmoset affected expression of genes in the ACC of adolescent monkeys (Law et al., 2009a). Additionally, both human and primate studies revealed EARE affected cerebellum, with human studies showing decreased cerebellum (Bauer et al., 2009; Edmiston et al., 2011), while a primate study revealing larger cerebellar vermis area in PR juvenile monkeys (Spinelli et al., 2009). EARE effect on primate cerebellum might due to the fact that macaque cerebellum has high density of glucocorticoid receptors (GRs) (Sánchez et al., 2000), which put it particularly vulnerable to stress hormones related over stimulation. Striatum was another brain region affected by EARE, with increased response to potential rewards (Casement et al., 2014) and elevated dopamine responses to amphetamine (Oswald et al., 2014), and a potential neurobiological mechanism linking early-life adversity and altered ventral striatal development was indicated (Goff & Tottenham, 2015). In addition to those specific regional changes, PR chimpanzees showed less global white-to-grey matter volume and cortical folding (Bogart et al., 2014). Structural connectivity between different brain regions was also affected by EARE, as studies showed affected corpus callosum, a wide and flat bundle of axons beneath the cortex connecting left and right cerebral hemispheres and facilitating inter-hemispheric communication, in a inconsistent way that most human studies showing EARE reduced corpus callosum (De Bellis et al., 1999; Rinne-Albers et al., 2016; Teicher et al., 2004, 1997), while few showing no significant changes (Mehta et al., 2009). Primate studies also found either decreased corpus callosum size (Sánchez et al., 1998) or no significant changes (Spinelli et al., 2009). 

### Neurons and glia cells

Neurons are the basic unit of brain. Neuronal network is responsible for the daily cognitive and emotional behaviors. Glia cell is a group of non-neuronal cells that support and protect the neurons in the brain. Rodent studies showed that maternal separation could induce morphological alteration of the apical dendrites of CA3 pyramidal neurons (Kwak et al., 2008); could increase corticotropin releasing factor (CRF)-containing neurons in amygdala (Becker et al., 2007); and could decrease *in vivo* firing activity of amygdala neurons (Adams & Rosenkranz, 2016) and sex related neurogenesis (Oomen et al., 2009). Chronic stress could induce atrophy of dendrites in hippocampus of rats (Brunson et al., 2005; McEwen, 1999) and tree shrews (Magariños et al., 1996), and could induce hippocampal neuroplasticity changes (Fenoglio et al., 2006). Bartesaghi and colleagues used guinea-pig as animal model to investigate the effects of early isolation on neurons, and they found that early isolation could induce morphologic changes of neurons in entorhinal cortex and hippocampus (Bartesaghi et al., 2003a, b ; Bartesaghi & Serrai, 2001, 2004). Although primate studies found neuronal morphological changes in EARE exposed monkeys (Bryan & Riesen, 1989; Floeter & Greenough, 1979; Stell & Riesen, 1987; Struble & Riesen, 1978), these early findings were limited to cerebellum, somatosensory and motor cortex, with limited information on other important brain regions, such as hippocampus, amygdala and PFC. Recent studies showed that different environments could induce neuron plasticity changes in the key brain regions involved in learning and memory. Complex environment could enhance complexity of the dendritic tree and density of dendritic spine in hippocampus and PFC in monkeys (Kozorovitskiy et al., 2005). Early parental deprivation in the marmoset monkey could produce long-term changes in hippocampal expression of genes involved in synaptic plasticity and implicated in mood disorder (Law et al., 2009b). So these neuron morphological and plasticity changes might explain and account for how EARE take effects on cell level, and then further more leading to behavioral changes.

 As EARE effects on glia cells, rodent studies revealed that EARE could induce long-term changes of astrocyte density and numbers in many brain regions, including PFC, mPFC, hippocampus, cingulate cortex and amygdala (Leventopoulos et al., 2007), and could alter behavioral, autonomic and endocrine responses to environmental challenge (Musholt et al., 2009; Rüedi-Bettschen et al., 2006). Although there was no direct evidence pointing out that glia cell changes were responsible for those altered responses in rats, those studies at least suggested the involvement of glia cell in EARE induced effects. Moreover, human studies showed that glial cell depletion in many brain regions was related to mood disorders, as the number of glia cell was reduced in PFC of both major depressive disorder (MDD) and bipolar disorder (BD) patients (Öngür et al., 1998), in the amygdala of major depressive disorder patients (Bowley et al., 2002) and in anterior cingulate cortex of major depressive disorder and schizophrenia patients (Cotter et al., 2001). Considering the important trophic influence of glia on neurons, glia cell deficits induced by EARE could possibly be responsible for EARE effects on neurons and furthermore to abnormal behavioral function. If that is true, how does it happen? Rodent Studies showed that stress related hormone glucocorticoid receptors (GRs) were also expressed in glia cells (Bohn et al., 1991; Jung-Testas & Baulieu, 1998; Vielkind et al., 1990). Glucocorticoid is the product of the HPA axis, so EARE might take effects through its influence on stress related hormones, like glucocorticoid, and then exert influence on glia cells leading to various effects (Jauregui-Huerta et al., 2010). Indeed, *in vitro* and *in* *vivo* studies showed that glucocorticoids could influence gene expression in glia cells (Bohn et al., 1994; Kumar et al., 1985) and could regulate the concentration of glial fibrillary acidic proteins (O'Callaghan et al., 1989). By playing central roles in learning and memory, hippocampal astrocyte number was dose-dependently increased by corticosterone treatment (Bridges et al., 2008), and glial responses in hippocampus was also regulated by glucocorticoid through influencing gene expression (Nichols et al., 2005). However, few studies were performed to investigate this issue and EARE affected glia cell changes were link directly to behavioral outcomes without solid evidence. In primate studies, there are lack of evidence to support that EARE affects glia cell structural and functional changes, and furthermore, induces behavioral outcomes.

### Lateralisation

Some studies suggested that the influence of EARE on different brain hemisphere might be different, and different type of EARE might take effects differentially on the same brain structure. A human study found that the institutionalized children had greater right amygdala volume, while the left amygdala volume was smaller in the children experienced longer periods of deprivation (Mehta et al., 2009). Another human study showed that patients with child abuse-related complex PTSD showed reduced gray matter concentration in right hippocampus and right dorsal ACC, but not in the left areas (Thomaes et al., 2010). In primate studies, maternal separation was associated with activation in the right dorsolateral PFC and decreased activity in the left dorsolateral PFC of juvenile rhesus monkeys (Rilling et al., 2001). Not only brain structure and function showed lateralisation affection by early experiences, behavioral research also found lateralisation in primates, as an interesting research showed that PR monkeys demonstrated greater left-hand bias compared to MR reared monkeys (Bennett et al., 2008). The number of lateralisation related studies is limited and the underlying mechanism remains unknown, which certainly adds complexity to the understanding of the influence EARE on brain structure and functional changes and the related abnormal behavioral outcomes.

### Other EARE effects

#### Young animals are particularly vulnerable to EARE effects

Adverse experience has its influence over all life stages, including early, middle and later life, in which infants are especially vulnerable to EARE and the consequences could persistent into later life. That might due to the fact that the most sensitive period of the whole life is the early stage, during which the body is undergoing profound physiological development, such as HPA axis, and brain is also undergoing profound neural development, such as neurogenesis. The amygdala developes rapidly during the early postnatal period in animals, e.g., in rats, cats and primates (Kikusui & Mori, 2009; Lupien et al., 2009; Payne et al., 2010; Wakefield & Levine, 1985). Stress related hormones and receptors were also maximally expressed in the brain early in development (Avishai-Eliner et al., 1996; Baram & Hatalski, 1998; Meaney & Szyf, 2005; Pryce et al., 2005a; Vazquez et al., 2006). These early physiological development heighten the vulnerability of the brain to environmental exposures. On the other hand, the proper development needs proper environmental stimuli, and the natural and best stimuli during early life is the attachment between caregivers, especially mothers, and infants, as mothers could supply tactile contact, physical warmth, nourishment, and psychological comforts. As stated in attachment theory and affectional system, infants need to develop a stable relationship with the mother for social and emotional development to occur normally, while various EARE intervene the forming of the bonds of this relation, therefore both short-term and long-term devastating influence are inevitable. 

#### Sexual differences in EARE effects

Humans studies showed that affectability of various mental disorders were sex-related during development, with boys showing higher tendencies to develop aggression and novelty seeking behaviors (Farrington & Loeber, 2000) while girls more susceptible to anxiety and depression (Kessler, 2003). Additionally, EARE influence might also be sex related, e.g., corpus callosum volume reduction was only found in EARE exposed males (De Bellis et al., 1999). Similarly, animal studies also revealed the vulnerability of males to EARE in rodents (Galea et al., 1997; Kikusui & Mori, 2009) and primates (Clarke, 1993; Cross & Harlow, 1965; Mitchell, 1968; Rommeck et al., 2009a; Suomi et al., 1971). On the contrary, other studies showed preference of EARE on females in humans (Heim & Nemeroff, 2001; Klimes-Dougan et al., 2001), rodents (Hoyer et al., 2013; Ziabreva et al., 2003b) and primates (Sánchez et al., 2005). Previous studies showed that stress could induce decreasing in number and length of apical dendritic branch of medial prefrontal cortex in male rats, whereas increasing in apical dendritic length in female rats (Garrett & Wellman, 2009). Isolated males showed less dendritic branches, shorter dendritic length and smaller dendritic spine density than control males, while isolated females had more dendritic branches than control females in guinea pig (Bartesaghi et al., 2003a). Neurogenesis was significantly increased in male but decreased in female offspring after maternal deprivation in rats (Oomen et al., 2009). The mechanism of those sexual differences remains unclear, but one possible explanation is the gender related physiological differences, such as neuroendocrine system and brain structure and function, which may induce different behavioral and physiological responses in male and female subjects. .

#### Time effects of EARE

Early life is a time of heightened susceptibility to EARE and expression of adverse experiences induced effects would be different across life time, therefore the time of administration of adverse experiences and subjects age of measurement might partially explain the discrepant findings across studies (Tottenham & Sheridan, 2009).

The time of adverse experiences administration is important, as different brain regions might have unique windows of vulnerability to stress, e.g., human studies indicate that the time window of hippocampus, corpus callosum and frontal cortex is at ages of 3-5, 9-10 and 14-16 years, respectively (Andersen et al., 2008). Rodent studies revealed the critical importance of specific time windows early in life for the outcome of maternal separation (Bock et al., 2005; Gos et al., 2008; Pryce et al., 2005b). Early primate studies by Harlow et al. showed the importance of administration time of adverse experiences (isolation), in a way that isolation beginning at birth generated most severe effects and persisting abnormalities (Harlow et al., 1965; Mitchell, 1968), while the isolation starting until later in life would produce less severe effects and persistent abnormalities (Harlow et al., 1965; Mitchell, 1968). Moreover, different lasting period of EARE also produced different effects even was all initiated at birth, i.e., 3 months isolation only induced reversible debilitating behavioral deficits, while at least six months isolation generated most severe effects and persisting abnormalities; 3 months isolation induced least, 6 months isolation induced moderate and 12 months isolation induced most severe defects (Griffin & Harlow, 1966; Harlow et al., 1965; Mitchell, 1968). These studies suggested that both the time point of administration of EARE and the lasting period have different influences on behavioral and biological outcomes.

Human studies showed different, or even contrary effects of EARE in children and adults, suggesting EARE might induce differential outcomes across lifespan. For example, childhood abuse induced significant reduction of hippocampal volume in adults (Bremner et al., 1997; Cohen et al., 2006; Stein et al., 1997; Woon & Hedges, 2008) but not in children (Carrion et al., 2001; De Bellis et al., 2001, 1999; Woon & Hedges, 2008); EARE induced hypercortisolism in children (Essex et al., 2002; Fernald & Gunnar, 2009; Flinn & England, 1997; Kaufman et al., 1997) but hypocortisolism in adults (Carpenter et al., 2009; Elzinga et al., 2008); adults with abuse related PTSD showed ACC volume reductions (Kitayama et al., 2006; Thomaes et al., 2010), whereas pediatric PTSD showed increased ACC (Richert et al., 2006). Primate studies also found similar results, e.g., monkeys exposed to EARE showed less aggression during infancy (Chamove et al., 1973; Harlow et al., 1965) but more aggression during latter life (Chamove et al., 1973; Mitchell, 1968), whereas showed more affiliative behavior during infancy (Chamove et al., 1973; Rosenblum & Paully, 1984; Ruppenthal et al., 1991) but less during adulthood (Kraemer & Mckinney, 1979; Rosenblum & Paully, 1984; Winslow et al., 2003). Monkeys exposed to EARE showed more activity during infants (Champoux et al., 1991) but less activity during adulthood (Harlow & Suomi, 1971a; Mason & Sponholz, 1963; Mitchell, 1968); The number and style of stereotypies exhibited in monkeys also varied by age, e.g., the number of whole-body stereotypies were negatively correlated with age, whereas self-directed stereotypies were positively correlated; moreover younger monkeys exhibited more pacing, body-flipping, and swinging, while older ones exhibited more hair-pulling and saluting (Lutz et al., 2003). These studies showed different, or even opposite effects of EARE on behavioral and biological outcomes between infants and adults, indicating EARE induce different outcomes across lifespan.

## MECHANISMS UNDERLYING EARE INDUCED EFFECTS

### Neuroendocrinological mechanisms

Some recent study linked behavioral outcomes with EARE affected neuroendocrine systems, and suggested that EARE might modulate subsequent social behaviors through regulating both the production and body's sensitivity to neurotransmitters and hormones (Cushing & Kramer, 2005). Moreover, studies indicate that the involved neurotransmitters and hormones were mainly monoamine neurotransmitter serotonergic systems, including serotonergic system and catecholamine system (both noradrenergic system and dopaminergic system), and glucocorticoid hormones (cortisol in non-human primates and humans), oxytocin and growth hormone (GH)(Table 4).

**Table 4:** Diet composition of *P. chungtienensis* and weatherfishes in Bita Lake

	Outcome		Primate studies	Human studies
Serotonin system	PR and SPR monkeys showed decreased CSF levels of 5-HIAA		Fahlke et al., 2000; Higley et al., 1996a; Maestripieri et al., 2006; Shannon et al., 2005	
	PR monkeys showed decreased SERT binding potential		Ichise et al., 2006	
	VFD monkeys were hyporesponsive to the serotonergic probe mCPP		Rosenblum et al., 1994	
Catecholamine system	PR monkeys showed lower CSF concentrations of HVA and attenuated NE secretion		Clarke et al., 1999; Clarke et al., 1996	
	VFD monkeys were hyper responsive to the noredrenergic probe yohimbine		Rosenblum et al., 1994	
	PR monkeys showed significantly lower DOPAC concentrations		Clarke et al., 1999; Clarke et al., 1996	
HPA axis dysregulation	Hypercortisolism	Hormone level	Barrett et al., 2009; Coplan et al., 2005; Coplan et al., 1996; Coplan et al., 2001; Suomi, 1991	Essex et al., 2002; Flinn & England, 1997; Gunnar et al., 2001; Kaufman et al., 1997
		Increased HPA response	Fahlke et al., 2000; Higley et al., 1992; Kraemer et al., 1983, 1984; Sánchez et al., 2005; Suomi, 1991	Heim et al., 2000b; Kikusui & Mori, 2009; Pesonen et al., 2010
	Hypocortisolism	Hormone level	Clarke et al., 1998; Capitanio et al., 2005; Shannon et al., 1998	Brand et al., 2010; De Bellis et al., 1994; Heim et al., 2000a
		Decreased HPA response	Barr et al., 2004; Capitanio et al., 2005; Clarke, 1993; Dettling et al., 1998; Dettling et al., 2002a, b ; Lyons et al., 2000; Parker et al., 2004	Carpenter et al., 2009; Elzinga et al., 2008; Hart et al., 1995

### Monoamine and hormone systems

The serotonergic system was shown to moderate the effects of EARE on the risk of depression in humans (Eley et al., 2004; Kaufman et al., 2004), and primate studies also indicate the role of serotonin system in regulating the effects of EARE. Maternal rejected, PR and SPR reared infant monkeys exhibited lower CSF 5-HIAA concentrations (Fahlke et al., 2000; Higley et al., 1996a; Maestripieri et al., 2006; Shannon et al., 2005); PR monkeys showed decreased SERT binding potential across a range of brain areas (Ichise et al., 2006); VFD reared monkeys were hyporesponsive to the serotonergic probe meta-Chlorophenylpiperazine (mCPP) (Rosenblum et al., 1994). Moreover, epigenetic studies also indicate the role of serotonin system plays in EARE induced HPA axis dysfunction (Barr et al., 2004; Rosenblum et al., 1994; Shannon et al., 2005; Spinelli et al., 2007) and subsequent abnormal behavioral outcomes (Barr et al., 2003, 2004; Law et al., 2009b; Maestripieri et al., 2006; Vicentic et al., 2006; Ziabreva et al., 2003a). Many studies showed catecholamine system is another candidate through which EARE takes its effect. PR monkeys showed attenuated Norepinephrine (NE) secretion (Clarke et al., 1999, 1996) and reduced CSF concentrations of catecholamine metabolite (Clarke et al., 1999, 1996), while VFD reared monkeys were hyper responsive to the noredrenergic probe yohimbine (Rosenblum et al., 1994). It was further suggested that EARE might influence the differentiation of noradrenergic neurons and thus alter HPA responses stress during adulthood (Liu et al., 2000). Dopamine system (another catecholamine system) might also be involved in EARE effects, as significantly lower concentrations of dopamine metabolite were revealed in PR infant monkeys (Clarke et al., 1999, 1996) and history of childhood adversity was positively associated with striatal dopamine responses to amphetamine (Oswald et al., 2014). 

Additionally, primate studies indicate potential hormonal pathways through which EARE takes effects, including oxytocin, growth hormone (GH), and most importantly, cortisol. Monkeys exposed to EARE showed abnormal aggressive and affiliative behaviors, and oxytocin was suggested to be a neuropeptide for affiliation and involved in the regulation of social bonding behaviors (Insel, 1992; Lim & Young, 2006). Therefore, oxytocin is a possible pathway for EARE to take effects, which indeed was probed by Winslow et al. (Winslow et al., 2003), showing that the decrease in affiliative behavior in PR rhesus monkeys was significantly and positively correlated with cerebrospinal oxytocin. Another hormone, GH, was also related to early adversity, as PR and social separation experiences in infant monkeys showed abnormal GH levels (Champoux et al., 1989a; Laudenslager et al., 1995). Most importantly, the main target of EARE under investigation is HPA axis. While some studies showed blunt HPA response, and thus decreased cortisol and ACTH levels (Barr et al., 2004; Capitanio et al., 2005; Clarke, 1993; Dettling et al., 1998, 2002a, b ; Lyons et al., 2000; Parker et al., 2004), others showed the opposite (Barrett et al., 2009; Coplan et al., 2005, 1996, 2001; Suomi, 1991). Although consistent results were not achieved, the importance of HPA dysregulation in EARE induced effects was suggested. 

### Hypothalamic-pituitary-adrenal (HPA) axis dysregulation

EARE is associated with elevated levels of stress and fear. The adverse impact of stress on brain development was suggested to be largely through hypothalamic-pituitary-adrenal (HPA) axis both in humans (Loman & Gunnar, 2010) and primates (Sanchez, 2006). The effects of EARE on HPA circadian rhythmicity and the function of HPA axis were reviewed in this section.

### Circadian rhythmicity

TheHPA axis is a complex set of direct influences and feedback interactions among three endocrine glands, i.e., hypothalamus, pituitary gland, and adrenal glands. Under basal conditions, HPA axis exhibits a circadian rhythmicity with a peak around the time of waking and a trough during the quiescent time of the activity cycle (Dickmeis et al., 2013; Leliavski et al., 2015; Tsang et al., 2016, 2014). So cortisol levels typically follow the circadian rhythm with levels highest occurring about 20 minutes after awakening in the morning (cortisol awakening response, CAR) and declining throughout the day. Alterations in the normal pattern of HPA rhythmicity, including CAR response and diurnal decrease of cortisol, were found in human studies. Most studies found higher morning cortisol level than controls in maltreated children (Cicchetti & Rogosch, 2001; Cutuli et al., 2010) and EARE exposed adults (Gonzalez et al., 2009; Gustafsson et al., 2010), while some found lower morning cortisol level (Carlson & Earls, 1997). Moreover, different kind of EARE might have differential influence on morning cortisol values, as studies indicate more emotionally and sexually abused children showed higher morning cortisol values, whereas more severe physically neglected and abused children showed lower levels (Bruce et al., 2009; Cicchetti & Rogosch, 2001). Additionally, EARE exposed children also showed higher incidences of atypical diurnal rhythmicity patterns, such as a peaking in the afternoon or evening (Cicchetti et al., 2010; Dozier et al., 2006). Similarly, abnormal HPA circadian rhythmicity were also found in limited amount of primate studies on rhesus monkeys, with morning peak occurring late in PR infants (Thomas et al., 1995) and flattened diurnal rhythm in repetitive maternal separation exposed infants (Sánchez et al., 2005). However, a recent study found no shift in diurnal patterns of cortisol in PR reared juvenile rhesus monkeys (Barrett et al., 2009). Although how EARE induces those abnormal HPA axis circadian rhythmicity, and its different or even contrary effects remains unknown, these HPA axis circadian rhythmicity abnormalities certainly contribute to various abnormal behavioral outcomes.

### HPA axis dysregulation

In humans, the HPA axis develops over the initial several years of life and is highly sensitive to EARE (De Weerth et al., 2003; Watamura et al., 2004). The key elements of the HPA axis are as following: the hypothalamus synthesizes and secretes corticotropin-releasing hormone (CRH); CRH stimulates the secretion of adrenocorticotropic hormone (ACTH) in pituitary gland; ACTH acts on the adrenal cortices, which then produces glucocorticoid hormones (mainly cortisol in NHPs and humans); glucocorticoids in turn act back on the hypothalamus and pituitary to suppress CRH and ACTH production in a negative feedback cycle. When activated in response to a stressor, the HPA axis participates in a cascade of neuroendocrine responses, and a typical HPA stress response involves a period of increased glucocorticoids in circulation induced by stimulation of elevated levels of CRH and ACTH, followed by a return to baseline levels induced by negative feedback of glucocorticoids (Herman & Cullinan, 1997). Thus CRH, ACTH and glucocorticoids levels could indicate the reactivity levels of HPA axis, with CRH an important neurotransmitter in HPA axis to initiate the autonomic and behavioral changes in response to stress (Heinrichs et al., 1995; Krohg et al., 2008; Ohmura & Yoshioka, 2009; Smagin & Dunn, 2000). Studies showed EARE induced elevated cerebrospinal fluid (CSF) concentrations of CRH levels in mother deprived rats (Ladd et al., 1996) and VFD reared infant monkeys (Coplan et al., 1996). Not only the CRH levels was increased, a study showed that EARE increased the density of CRH binding sites in many brain regions, including PFC cortex, amygdala and hippocampus (Anisman et al., 1998). As analysis of CRH requires sampling of CSF, it was hard to perform the experiment on healthy humans. On the other hand, analysis of ACTH and glucocorticoids (cortisol) only requires blood or urine sampling, so they are more widely investigated in humans. 

Glucocorticoids was revealed to be released from the adrenal cortex during neuroendocrine responses to stress (Herman et al., 2003, 1996), and then regulate HPA axis via negative feedback by binding to two types of receptors, mineralocorticoid receptors (MRs) with high affinity (important in proactive maintenance of HPA basal activity), and GRs with low affinity (primarily responsible for negative feedback). Glucocorticoids could pass through the blood-brain barrier to influence brain function (Zarrow et al., 1970), and MRs' expression was significantly greater in monkey infants than other ages (Pryce et al., 2005a). Therefore, HPA axis was one of the major pathways through which EARE induces stress and shapes brain development, particular in infants. Glucocorticoids could facilitate HPA axis activation by occupying its receptors in amygdala, leading CRH increase within amygdala (Kolber et al., 2008), whereas it could also suppress HPA axis by occupying its hippocampal receptors (van Haarst et al., 1997). Amygdala and hippocampus are important brain regions for socio-emotional functioning and learning and memory throughout development, and they have a high density of receptors for unbound glucocorticoids, therefore are major targets of EARE affected HPA axis (Johnson et al., 2005; Sánchez et al., 2000). Additionally, EARE could affect HPA axis function bidirectionally, with some studies showing attenuated basal and challenge induced levels of cortisol (hypocortisolism), while others showing elevated levels in both conditions (hypercortisolism).

### Hypercortisolism

Human studies showed that EARE could induce hypercortisolism of basal cortisol level in children, reflected by elevated levels of cortisol (Essex et al., 2002; Flinn & England, 1997; Gunnar et al., 2001) and ACTH (Kaufman et al., 1997) in EARE exposed children. Primate studies also showed EARE induced hypercortisolism, reflected by increased plasma coritsol and ACTH in PR infants and juvenile monkeys (Barrett et al., 2009; Suomi, 1991). The elevated cortisol levels in hairs of PR infants indicate the long time accumulation of EARE outcomes (Dettmer et al., 2012). Increased basal cortisol levels were found to be induced by prenatal stress (Pryce et al., 2011). Persistently elevated CSF concentrations of CRF in both infants and mothers under VFD conditions were reported (Coplan et al., 2005, 1996). Not only EARE could affect infants and children, it was suggested that childhood abuse was associated with a persistent sensitization of the HPA axis to stress in human adults (Elzinga et al., 2008), e.g., adults exposed to EARE had higher HPA reactivity during the Trier Social Stress Test (TSST) (Heim et al., 2000b; Pesonen et al., 2010). Animal studies also showed hyper-response of HPA axis activity when facing stress in both infants and adults exposed to EARE. Rodent studies revealed higher HPA response to novelty stress in early-weaned mice (Kikusui & Mori, 2009). Primate studies showed hyper-responsiveness in EARE reared monkeys, reflected by increased cortisol response to stress in monkeys exposed to PR rearing (Fahlke et al., 2000; Suomi, 1991), VFD rearing (Coplan et al., 2001), repetitive maternal separation (Sánchez et al., 2005) and parental deprivation (Higley et al., 1992; Sánchez et al., 2005). Amphetamine challenge test also revealed neurochemical and behavioral hyper-responseness in isolated monkekys (Kraemer et al., 1983, 1984). All those studies suggested EARE induced hypercortisolism, reflected by elevated basal and stress or challenge facing levels of cortisol, ACTH or CRF.

### Hypocortisolism

EARE induced hypocortisolism was also a common finding (Gunnar & Vazquez, 2001), e.g., maltreated children showed decreased basal levels of cortisol (Brand et al., 2010; Heim et al., 2000a) and ACTH (De Bellis et al., 1994). Primate studies revealed attenuated basal levels of cortisol and ACTH in PR monkeys (Capitanio et al., 2005; Clarke et al., 1998; Shannon et al., 1998). When facing stress or challenge, EARE exposed children and adults both showed blunt cortisol response and thus reduced cortisol level (Carpenter et al., 2009; Elzinga et al., 2008; Hart et al., 1995). Similarly, primate studies showed blunt HPA responses during stress and thus decreased coritsol and ACTH levels in PR reared infants (Barr et al., 2004; Capitanio et al., 2005; Clarke, 1993), in young adults exposed to maternal deprivation and intermittent separation (Capitanio et al., 2005; Lyons et al., 2000) and in the hairs of PR infants (Feng et al., 2011) of rhesus monkeys. EARE induced hypocortisolism was also found in other monkey species, including maternal neglect exposed juvenile Goeldi's monkeys (Dettling et al., 1998), intermittent stress exposed squirrel monkeys (Parker et al., 2004) and parental deprivation exposed marmosets (Dettling et al., 2002a, b ). All those studies indicate hypocortisolism reflected by decreased basal and stress or challenge facing levels of cortisol or ACTH.

As described above, it is controversial as to the effects of EARE on HPA axis, with some studies showing hypercortisolism while other showing hypocortisolism. There are several possible reasons. Firstly, different types of EARE vary between different research, and even for a same type of EARE, the procedures, manipulations, tests and measuring indexes could be different in different experiments. Secondly, different genotype among human races or animal species could contribute to the divergence as well, in a way that individuals with certain genotype may be more sensitive to a particular type of EARE than others. In addition, subjects' personality or temperaments could also partially contribute to the divergence, e.g., children with inhibited temperaments tended to have higher cortisol levels than extroverted children (Gunnar et al., 1995; Kagan et al., 1988), indicating that long-term consequences of EARE may not uniform across subject populations..

### A sample of EARE induced neurotransmitter and hormonal changes related behavioral outcomes - social status of primates

Studies suggest that EARE could induce abnormal changes of neurotransmitters and hormones and then influence social status of primates. Serotonergic system was the most widely studied neurotransmitter involved. Primate studies showed that different levels of CSF serotonin (5-HT) or its main metabolite 5-Hydroxyindoleacetic acid (5HIAA) were related to different social status, with higher levels related to more dominant status (Higley et al., 1996b; Raleigh et al., 1983). Additionally, serotonergic drugs were found to be able to influence dominance status, in a way that serotonergic enhancing drugs increase social dominance while serotonergic reducing drugs decrease dominance (Raleigh et al., 1991). 5-HT seems to be positively related to social dominance status, and studies suggested that might due to its influence on affiliative and aggressive behaviors which are important factors in dominance formation and maintenance. Primate studies showed positive correlation between CSF 5-HIAA and affiliative approaching and grooming behavior (Mehlman et al., 1995; Raleigh et al., 1985) and negative correlation between CSF 5-HIAA and aggressive behavior (Higley et al., 1996a), which were consistent with the previous presumption that affliliative behavior was much more effective in acquiring and reinforcing social dominance than aggressive behaviors. Supporting evidence also came from another genetic primate study that suggested certain serotonin transporter (5-HTT/SERT) diplotypes might modulate acquisition of dominance status (Miller-Butterworth et al., 2007). Beside serotonergic system, dopaminergic systems might also affect social dominance status, as dopamine transporter (DAT) gene variants were suggested to be associated with social rank in cynomolgus monkeys (Miller-Butterworth et al., 2008). As to hormones, although a study showed that cortisol concentration was significantly higher in dominant monkeys (Czoty et al., 2009), most studies failed to find the relationship between cortisol level and social rank (Czoty et al., 2009; Goo & Sassenrath, 1980; Morgan et al., 2000; Stavisky et al., 2001). Those studies suggested that EARE could influence social status of primates through its influence on neurotransmitters and hormones. The effects of EARE should not be limited on social status but also might on some other abnormal behaviors.

### Genetic and epigenetic influences of EARE effects

Developing is a dynamic process involving constant and reciprocal interactions between organisms and the environments. Emerging evidence suggests that epigenetic modifications may serve as a critical mechanism through which experiences occurring during the lifespan can have sustained effects in developmental outcomes (Daskalakis et al., 2013). Epigenetics refers to the study of inherited changes in phenotype (appearance) or gene expression caused by mechanisms other than changes in the underlying DNA sequence, such as modifications of transcription of the genome by chemical markers regulation, and variation in gene expression rather than gene sequence is the key concept. Moreover, epigenetics is used to describe the dynamic interactions between genome and the environment (Jablonka & Lamb, 2002). Research suggested that environmental events can modify the epigenetic status of the genome by activating intracellular pathways to regulate interaction between transcription factors and their DNA binding sites, leading to changes in gene expression and eventually different levels of proteins (Bagot & Meaney, 2010; Zhang & Meaney, 2010). This is the biological basis for the interplay between environmental factors and the genome in the regulation of individual differences in behaviors and cognition. Both animal and human studies suggest that EARE can lead to lasting changes in neurotransmitter systems and brain function, and then induce cognitive and behavioral changes. However, there was remarked inter-individual variations in responses to adversity (Collishaw et al., 2007; Rutter, 2007), and these variations might be due to different genotype, different living environment and interaction between the genome and environment.

### Genetic influences

Different genotype could induce different behavioral outcomes. Allelic variation of the monoamine oxidase A (MAOA) gene was implicated in aggressive behaviors (Volavka et al., 2004). Both human (Caspi et al., 2002; Craig, 2005; Kim-Cohen et al., 2006) and primate (Karere et al., 2009) studies showed that genotype conferring low MAOA activity was related to mental health problems. These findings may partially explain the variability in developmental outcomes associated with maltreatment, e.g., why not all victims of maltreatment grow up to with abnormal behaviors like antisocial problems, and they provide epidemiological evidence that genotypes can moderate children's sensitivity to environmental insults. Similar results was revealed in 5-HTT genotype, as short promoter region of the serotonin transporter (5-HTTLPR) allele was related to increased anxious behavior in primates (McCormack et al., 2009) and highest emotional problem scores in human (Kumsta et al., 2010). Those evidences suggest the importance of genotype in behavioral outcomes.

### Epigenetic influences of EARE on gene expression

Environmental and life experience could exert influences on gene expression and time course analysis indicate that maternally induced epigenetics might emerge during the postnatal period and could sustain into adulthood (Weaver et al., 2004). Epigenetic regulation of gene expression is particularly important during the early stages of development, and it is one of the main mechanisms mediating the long-term effects of maternal care on development (Champagne, 2008; Champagne & Curley, 2009; Diorio & Meaney, 2007; Meaney, 2001; Zhang et al., 2006). For example, rodent studies showed that postnatal maternal licking/grooming (LG) behavior could induce increased hippocampal GR expression (Caldji et al., 1998; Francis et al., 1999; Liu et al., 1997; Weaver, 2007), while low levels of LG during neonates led to reduced expression of estrogen receptor in hypothalamus and reduced response to estrogen (Champagne et al., 2001, 2006, 2003). As to the effects of EARE on gene expression, isolation attenuated social interaction induced gene expression in rodents (Ahern et al., 2016; Lukkes et al., 2012, 2013; Shishkina et al., 2015; Wall et al., 2012). A human study also showed EARE related down regulation of genes containing GR response elements (Miller et al., 2009). In primate studies, early maternal separation could lead to gene expression changes in many brain regions, including differential expression of gene GUCY1A3 in amygdala, decreases in hippocampal growth associated protein 43 (GAP-43) mRNA and 5-HT receptor mRNA (Law et al., 2009b) and a selective long-term effect on expression of genes in ACC (Law et al., 2009a). Moreover, epigenetics is not a binary response across the whole brain. Different genes in different brain regions can be affected in different ways, e.g., early maternal deprivation could either induce reduction of gene expression (Liu et al., 1997; Roceri et al., 2002) or up-regulation of gene expressions (Plotsky et al., 2005; Ziabreva et al., 2000). 

### Epigenetic influences of EARE on neurobiological and behavioral outcomes

Epigenetic influences on gene expression may lead to different expression patterns of proteins, thus different levels of hormones and neurotransmitters, ultimately lead to different behavioral outcomes. Studies suggested that EARE might exert its effects on behavioral outcomes independent of genotype. Primate studies revealed the importance of environment and life experience independent of genotype. MR monkeys showed significantly up-regulated level of 5-HTT during maternal separation, while NR monkeys did not (Kinnally et al., 2008). With the same low-activity Monoamine oxidase A (MAOA) genotype, MR reared monkeys were more aggressive than the PR monkeys (Newman et al., 2005). Higher 5-HTT cytosine-phosphate-guanosine (CpG) methylation, but not rh5-HTTLPR genotype, exacerbated the effects of early life stress on behavioral stress reactivity in infant monkeys (Kinnally et al., 2010). Additionally, from behavioral perspective alone, infant monkeys exposed to mother abuse showed the same tendency to their offspring, regardless of whether they were reared by their biological mothers or by foster mothers (Maestripieri, 2005). Rodent studies also supported the important role of environment and life experience on behavioral outcomes. Rodent studies suggested that maternal care behaviors especially postnatal maternal LG could be transmitted from the mother to her female offspring, so female offspring who received low levels of LG also provided low levels of this form of maternal care to their own offspring (Fleming et al., 2002). Cross-fostering studies in rodents indicate that this intergenerational transmission of behaviors was the result of early experience rather than genetic inheritance (Champagne & Meaney, 2001). For example, the biological offspring of low-LG mothers reared by high-LG dams resembled the normal offspring of high-LG mothers (Francis et al., 1999). All those studies indicate the importance of environment and life experience, especially maternal interactions on the subsequent expression of behaviors, rather than the genetic contributions. Moreover, primate studies showed the importance of the interaction between genetic factors and environmental experience on neurobiological outcomes, as CSF 5-HIAA concentrations were significantly influenced by genotype in the PR but not MR reared monkeys (Bennett et al., 2002), and 5-HTT gene variation affected HPA axis activity in response to stress in a way that cortisol levels increased during separation in MR but decreased in PR monkeys (Barr et al., 2004). 

### Transgenerational epigenetic programming

Among the epigenetic mechanisms of EARE, such as DNA methylation, histone modifications, and micro-RNA expression, DNA methylation was the most intensively studied epigenetic phenomenon (Babenko et al., 2015; Blaze et al., 2015b; Jawahar et al., 2015; Provençal et al., 2015; Vaiserman, 2015a, b ; Zheng et al., 2014). Actually, not only EARE exposure of the offspring themselves could lead to long time biological and behavioral outcomes, EARE exposure of parents could also influence their offspring, which was defined as transgenerational epigenetic programming phenomenon and has drawn much attention recently. Related studies suggested the underlying epigenetic mechanisms of maternal transgenerational influence to be DNA methylation, histone modifications, and micro-RNA expression (Babenko et al., 2015; Bale, 2014; Blaze & Roth, 2015; Gröger et al., 2016; Miska & Ferguson-Smith, 2016; Nagy & Turecki, 2015).

In addition to maternal influences, fathers can exert influences on offspring development either through direct care in living social environment, or indirectly through interacting with maternal influences. Some recent studies showed the important influence of paternal early experiences on infant development through non-social mechanisms, even in the absence of direct contact with offspring. This emerging field focuses on how environmental influences can epigenetically alter paternal sperm DNA methylation, histone modification and micro-RNA expression, and ultimately change the phenotype and behavior of offspring (Braun & Champagne, 2014; Curley et al., 2011; Day et al., 2016; Kinnally & Capitanio, 2015; Rodgers et al., 2013; Yuan et al., 2016). 

## DISCUSSION

### Rehabilitation

Human studies indicate the possibility of rehabilitation of EARE induced deficits, e.g., Fisher et al.(2000, 2006, 2007) suggested that the improvements of caring following EARE had the potential to prevent or reverse EARE induced HPA axis dysfunction, such as normalizing perturbed diurnal cortisol patterns and reducing basal salivary cortisol level (Fernald & Gunnar, 2009). In primate studies, total social isolation was once considered to induce permanent defect, which was described as learning deficit in some studies, because isolated monkeys were lack of physical interactions and had no opportunity for social learning with conspecifics, or to gradually develop sophisticated social behaviors (Mitchell, 1968; Sackett, 1969; Suomi et al., 1974). These abnormities might be rehabilitated by socializing the isolate monkeys with conspecifics. Some studies reported that after the isolated monkeys was paired with "therapist" monkeys, less self-directed disturbance activities, or stereotypic behaviors, but more social contact and exploratory behaviors were observed (Harlow & Suomi, 1971b; Suomi, 1973). Less severe self-injurious behaviors were found when isolated monkeys were reared with surrogates (Brunelli et al., 2014; Harlow & Suomi, 1971b) or social housing (Lutz & Novak, 2005). Additionally, environmental enrichment treatments were used to eliminate abnormal behaviors and to normalize the behavioral repertoire of EARE exposed monkeys (Lutz & Novak, 2005; Novak et al., 1998; Rommeck et al., 2009a). It was suggested that environmental enrichment devices could only ameliorate less severe forms of abnormal behavior but not more severe forms of self-injurious or non-injurious self-abuse behaviors (Rommeck et al., 2009a). Those studies indicate rehabilitation is possible, at least partially, but it requires combination of multiple rehabilitation methods, such as socializing, environmental enrichment, and considerable time and effort.

### Establishing NHP mental disorder models with EARE methods

EARE exposed NHPs showed signs of various mental disorders, including anxiety, autism and depression etc., making it a potential animal model to study human mental disorders, among which depression model was the mostly investigated one (Gilmer & McKinney, 2003; Pryce et al., 2005b; Worlein, 2014). The influence of EARE on NHPs is through daily life and accumulates over a period of time, making it a more natural model of mental disorders than those induced by drugs or invasive surgeries. The diagnosis of mental disorders in humans usually depends on questionnaire investigation and verbal communication between patients and doctors, which are not doable in NHPs. Therefore NHPs studies usually include daily group living or single subject observation and behavioral analysis, biochemical index analysis (e.g., hormones), brain structural and functional changes analysis by using modern imaging methods, However, due to the complexity of mental disorders, it is very difficult to diagnose mental disorders in NHPs. Different disorders might show very similar behavioral symptoms and biochemical abnormalities, therefore additional indexes are necessary in diagnosing. For example, in NHPs, anxiety and depression share symptoms of stereotypic behaviors and elevated cortisol level in response to stressor, so, more depression specific symptoms, such as lacking of responses to stimulus are needed for diagnosing. It is difficult to differentiate if a monkey was depressed or autistic as in both cases, preference of staying away from social activity and being alone in a corner would be shown. Certain metal disorders could be divided into many subtypes, e.g., depression includes unipolar, bipolar and atypical depression, etc, which certainly adds more complexity in establishing NHP animal models. These might explain the fact that despite being an ideal and irreplaceable animal model, studies on EARE induced NHP mental disorder models are limited. 

## CONCLUDING REMARKS

In summary, as an irreplaceable animal model, NHP EARE experiments were performed for over 60 years and revealed important insights into understanding the effects of EARE on development and underlying mechanisms of related physiological and psychological diseases. Although much has been learned to date, there is much more to understand about EARE impact on developmental trajectory. Now, with the help of emerging cutting edge technologies, such as new brain imaging method, gene modification, optogenetics, etc, future EARE studies will further clarify these issues and help to cure the diseases.
